# A tensor product quasi-Poisson model for estimating health effects of multiple ambient pollutants on mortality

**DOI:** 10.1186/s12940-019-0473-7

**Published:** 2019-04-24

**Authors:** Li-Jun Xu, Shuang-Quan Shen, Li Li, Ting-Ting Chen, Zhi-Ying Zhan, Chun-Quan Ou

**Affiliations:** State Key Laboratory of Organ Failure Research, Department of Biostatistics, Guangdong Provincial Key Laboratory of Tropical Disease Research, School of Public Health, Southern Medical University, Guangzhou, 510515 China

**Keywords:** Tensor product, Quasi-Poisson regression, Collinearity, Health effects

## Abstract

**Background:**

People are exposed to mixtures of highly correlated gaseous, liquid and solid pollutants. However, in previous studies, the assessment of air pollution effects was mainly based on single-pollutant models or was simultaneously included as multiple pollutants in a model. It is essential to develop appropriate methods to accurately estimate the health effects of multiple pollutants in the presence of a high correlation between pollutants.

**Methods:**

The flexible tensor product smooths of multiple pollutants was applied for the first time in a quasi-Poisson model to estimate the health effects of SO_2_, NO_2_ and PM_10_ on daily all-cause deaths during 2005–2012 in Guangzhou, China. The results were compared with those from three other conventional models, including the single-pollutant model and the three-pollutant model with and without first-order interactions.

**Results:**

The tensor product model revealed a complex interaction among three pollutants and significant combined effects of PM_10_, NO_2_ and SO_2_, which revealed a 2.53% (95%CI: 1.03–4.01%) increase in mortality associated with an interquartile-range (IQR) increase in the concentrations of all three pollutants. The combined effect estimated by the single-pollutant model was 5.63% (95% CI: 3.96–7.34%). Although the conventional three-pollutant models produced combined effect estimates (2.20, 95%CI, 1.18–3.23%; 2.78, 95%CI: 1.35–4.23%) similar to those of the tensor product model, they distorted the estimates and inflated the variances of the estimates when attributing the combined health effects to individual pollutants.

**Conclusions:**

The single-pollutant model or conventional multi-pollutant model may yield misleading results in the presence of collinearity. The tensor product quasi-Poisson regression provides a novel approach to the assessment of the health impacts of multiple pollutants by flexibly fitting the interaction effects and avoiding the collinearity problem.

**Electronic supplementary material:**

The online version of this article (10.1186/s12940-019-0473-7) contains supplementary material, which is available to authorized users.

## Background

An increasing number of studies have demonstrated the association between daily ambient air pollution levels and adverse health outcomes worldwide [1–4]. Some studies generally estimated the health effect of each pollutant separately via a single-pollutant model controlling for time trends and meteorological factors [5–7]. However, pollution occurs as a mixture of highly correlated gaseous, liquid and solid pollutants. Some studies observed that the effect using the single-pollutant model was weakened after other pollutants were simultaneously included [8]. Therefore, the results of the single-pollutant model tends to overestimate the health effects of pollutants. Moreover, many studies observed synergistic effects of combinations of pollutants on health outcomes [9, 10]. To advance our understanding of the biological mechanisms of pollutant toxicity, it is essential to move from the single-pollutant model to the multi-pollutant model [11]. This would provide important information for guiding regulatory policies for public health.

Many researchers have recognized the limitations of the single-pollutant model and have taken some efforts to overcome such limitations. One of commonly used methods is to simultaneously include two or three pollutant variables in a generalized additive model (GAM); then, the effects of one pollutant can be estimated after adjusting for other pollutants in the model [12–14]. This simple resolution ignores the high correlation of the occurrence of pollutants, which likely leads to a collinearity problem, such as variance inflation or bias even reverse estimation of parameters. For example, Chen et al. [15] and Kan et al. [16] showed that PM_10_ was negatively associated with daily respiratory mortality when the NO_2_ or SO_2_ was considered simultaneously, suggesting that the PM_10_ had a protective effect. To alleviate this problem, the Health Effects Institute supports the development of innovative statistical methods for studying the health effects of multiple pollutants in the presence of collinearity.

Several different approaches have been attempted to analyze the effects of multiple pollutants. One approach is the principal component analysis (PCA) that can avoid unstable parameter estimates in the condition of collinearity [17, 18]. However, PCA only maximizes the variance interpreted by the linear combination of independent variables without directly considering the relationship between the dependent variable and independent variables. Partial least-squares regression (PLSR) combining PCA with canonical correlation analysis captures not only the information of independent variables but also the relationship with the dependent variable, yet this approach produces linear combinations of the original independent variables that are difficult to interpret in practice. Using Monte Carlo simulations, Månsson and Shukur [19] showed that the estimators produced by Poisson ridge regression were more accurate (i.e. had less standard errors) than those produced by the Poisson log-linear model. However, the increase in accuracy was at the expense of increasing bias in the condition of strong collinearity. Bayesian model averaging (BMA) averages the effects of models for all possible combinations of independent variables; these models are weighted by the posterior probability of the model [20]. However, there are different views on the interpretation of effect estimates from BMA in the case of extensive collinearity. Bobb et al. [21] proposed Bayesian kernel machine regression (BKMR) that regressed the heath outcome on a flexible kernel function of multiple pollutants. BKMR can be highly sensitive to the specification of the prior distribution and can solve the problem of collinearity using hierarchical variable selection.

To date, there is no standard model for evaluating health effects of multiple pollutants. Sun et al. [22] concluded that no method was unanimous across all simulation scenarios that differed in sample size, the number of pollutants and the strength of exposure-response association. The choice of the appropriate method should depend on the goal of the study. Wood proposed a general method for constructing low-rank tensor product smooths of multiple variables that could quantitatively estimate the nonlinear effects of multiple independent variables on a dependent variable [23, 24]. The tensor product smooths has been widely applied in geometry, physics, mechanics and quantum theory [25]. To the best of our knowledge, the tensor product smooths for GAM was never employed to evaluate the health impacts of multiple pollutants. This study employed firstly this approach to assess non-linear effects and potential interaction effects of SO_2_, NO_2_ and PM_10_ on mortality in Guangzhou, China, during 2005–2012.

## Methods

### Data

Daily meteorological data for mean temperature, relative humidity and atmospheric pressure were collected at the Guangzhou Weather Station and were downloaded from the China Meteorological Data Sharing Service System. Air pollution data, including particulate matter < 10 μm in aerodynamic diameter (PM_10_), nitrogen dioxide (NO_2_) and sulfur dioxide (SO_2_), were provided by the Guangzhou Bureau of Environmental Protection. The daily concentrations of PM_10_, NO_2_ and SO_2_ were the average levels collected from seven fixed-site air quality monitoring stations that were all located in central urban areas and had complete data during the study period of 2005–2012 [26]. There were 40 missing observations for PM_10_, NO_2_, and SO_2_, accounting for 1.5% of the study days. We did not replace the missing values. Because the levels of air pollution and health effects of pollution on mortality may be different in suburban areas, to maintain consistency of the study population, the mortality data were also limited to death registration records of the whole population at six central urban districts.

### Statistical analysis

First, we constructed a basic quasi-Poisson regression model without air pollutant variables. Natural cubic splines (ns) of daily mean relative humidity, atmospheric pressure and temperature were used to control the nonlinear and non-monotonic confounding effects of meteorological factors on mortality. Time can be considered as a proxy of some other unobserved time-varying confounders. A natural cubic spline function of time was used to control for the long-term trend and seasonal variations of daily mortality and potential confounding effects of unobserved time-varying covariates. The partial autocorrelation function (PACF) of residuals and the Akaike’s Information Criterion (AIC) were used to guide the selection of the number degrees of freedom (df) for ns. When a df of 7 was used for time variables, the PACF was free of patterns and was not autocorrelated [16, 27]. The value of df equal to 3 was used for meteorological variables because this value has been shown to control well their impacts on daily mortality changes [28–30]. We used a 14-day moving average of mean temperature to control the lag effect because previous studies in Guangzhou observed that the effects of mean temperature on mortality persisted for approximately 14 days [31, 32]. Day of the week (DOW) and a holiday (HOD) indicator were included in the model as indicator variables.

After the basic model was constructed, we added air pollution variable(s) to the model. The reason was that many studies, including our data, showed that the adverse impacts of each specific pollutant were statistically significant at lag 0 and lag 1, and these impacts almost disappeared at lag 3 and during longer lags [33, 34]. To adequately capture the lag effects, a 3-day moving average of air pollution concentrations (i.e., lag 0–2) was used, and the concentrations were introduced into the model in the following four ways:

Model I (single-pollutant model) incorporated each of pollutant variables into the basic model, separately:

1 $$ {\displaystyle \begin{array}{l}\mathrm{Log}\left({\upmu}_t\right)=\alpha +{\boldsymbol{\uptheta}}_1\mathrm{ns}\left({\mathrm{Temperature}}_{\mathrm{t}}\right)+{\boldsymbol{\uptheta}}_2\mathrm{ns}\left({\mathrm{Humidity}}_{\mathrm{t}}\right)+{\boldsymbol{\uptheta}}_3\mathrm{ns}\left({\mathrm{Pressure}}_{\mathrm{t}}\right)\\ {}\kern0.5em +{\boldsymbol{\uptheta}}_4\mathrm{ns}\left(\mathrm{Time}\right)+{\delta}_1{\mathrm{DOW}}_t+{\delta}_2{\mathrm{HOD}}_t+\upbeta \left({\mathrm{PM}}_{10t}/{\mathrm{NO}}_{2\ t}/{\mathrm{SO}}_{2t}\right),\end{array}} $$

where β(PM_10*t*_/NO_2*t*_/SO_2*t*_) signifies that respective single-pollutant models for PM_10_, NO_2_ and SO_2_ are established; β is the coefficient of a pollutant variable.

Model II (three-pollutant non-interaction model), simultaneously included three pollutant variables without considering first-order interactions and is specified as follows:

2 $$ {\displaystyle \begin{array}{l}\mathrm{Log}\left({\upmu}_t\right)=\alpha +{\boldsymbol{\uptheta}}_1\mathrm{ns}\left({\mathrm{Temperature}}_{\mathrm{t}}\right)+{\boldsymbol{\uptheta}}_2\mathrm{ns}\left({\mathrm{Humidity}}_{\mathrm{t}}\right)+{\boldsymbol{\uptheta}}_3\mathrm{ns}\left({\mathrm{Pressure}}_{\mathrm{t}}\right)\\ {}+{\boldsymbol{\uptheta}}_4\mathrm{ns}\left(\mathrm{Time}\right)+{\delta}_1{\mathrm{DOW}}_t+{\delta}_2{\mathrm{HOD}}_t+{\beta}_1{\mathrm{PM}}_{10t}+{\beta}_2{\mathrm{NO}}_{2t}+{\beta}_3{\mathrm{SO}}_{2t}.\end{array}} $$

Model III (three-pollutant interaction model) adds linear terms of the first-order interaction between pollutants to model II:

3 $$ {\displaystyle \begin{array}{c}\mathrm{Log}\left({\upmu}_{\mathrm{t}}\right)=\alpha +{\boldsymbol{\uptheta}}_1\mathrm{ns}\left({\mathrm{Temperature}}_{\mathrm{t}}\right)+{\boldsymbol{\uptheta}}_2\mathrm{ns}\left({\mathrm{Humidity}}_{\mathrm{t}}\right)+{\boldsymbol{\uptheta}}_3\mathrm{ns}\left({\mathrm{Pressure}}_{\mathrm{t}}\right)\\ {}+{\boldsymbol{\uptheta}}_4\mathrm{ns}\left(\mathrm{Time}\right)+{\delta}_1{\mathrm{DOW}}_t+{\delta}_2{\mathrm{HOD}}_t+{\beta}_1{\mathrm{PM}}_{10t}+{\beta}_2{\mathrm{NO}}_{2t}+{\beta}_3{\mathrm{SO}}_{2t}\\ {}+{\beta}_{12}\left({\mathrm{PM}}_{10t}\times {\mathrm{NO}}_{2t}\right)+{\beta}_{13}\left({\mathrm{PM}}_{10t}\times {\mathrm{SO}}_{2t}\right)+{\beta}_{23}\left({\mathrm{NO}}_{2t}\times {\mathrm{SO}}_{2t}\right),\end{array}} $$

where *β*_1_, *β*_2_ and *β*_3_ are the regression coefficients of pollutants, and *β*_12_, *β*_13_ and *β*_23_ are the regression coefficients of first-order interaction terms.

Model IV (tensor product model) constructs the tensor product smooths (te) of three pollutants (PM_10_, NO_2_ and SO_2_)and is specified as follows:

4 $$ {\displaystyle \begin{array}{c}\mathrm{Log}\left({\upmu}_{\mathrm{t}}\right)=\alpha +{\boldsymbol{\uptheta}}_1\mathrm{ns}\left({\mathrm{Temperature}}_{\mathrm{t}}\right)+{\boldsymbol{\uptheta}}_2\mathrm{ns}\left({\mathrm{Humidity}}_{\mathrm{t}}\right)+{\boldsymbol{\uptheta}}_3\mathrm{ns}\left({\mathrm{Pressure}}_{\mathrm{t}}\right)\\ {}+{\boldsymbol{\uptheta}}_4\mathrm{ns}\left(\mathrm{Time}\right)+{\delta}_1{\mathrm{DOW}}_t+{\delta}_2{\mathrm{HOD}}_t+ te\left({\mathrm{PM}}_{10},{\mathrm{NO}}_2,{\mathrm{SO}}_2\right),\end{array}} $$

where *te*(PM_10_, NO_2_, SO_2_) is the tensor product smooth function of three pollutants. For models I-IV, μ_t_ is the expected number of deaths on day t, *α* is the intercept, ns(.) denotes the unknown smooth functions modeled by natural cubic splines, and **θ**_**1**_**- θ**_**4**_ represent the coefficients vector of smooth functions.

Construction of the tensor product smooths has been described previously in detail [23]. Mathematically, supposing that there are two vector spaces **V** and **W**, where the **V** has a basis *e*_*i*_ (*i* = 1 … *m*), and the W has a basis *f*_*j*_ (*j* = 1 … *n*), the tensor product of **V** ⊗ **W** is generated by $\sum \limits_{i=1}^m\sum \limits_{j=1}^n{e}_i\otimes {f}_j$, where the product operation ⊗ is Kronecker product. In this paper, we briefly introduce the construction process of tensor product smooths of three covariates (i.e., PM_10_, NO_2_, SO_2_). It is easy to generalize the tensor product smooths to more variables in the same way. Basis functions for representing the smooth function of each pollutant are given by $f\left({PM}_{10}\right)=\sum \limits_{i=1}^I{\alpha}_i{\phi}_i\left({\mathrm{PM}}_{10}\right)$, $f\left({\mathrm{NO}}_2\right)=\sum \limits_{k=1}^K{\omega}_k{\eta}_k\left({\mathrm{NO}}_2\right)$ and $f\left({\mathrm{SO}}_2\right)=\sum \limits_{l=1}^L{b}_l{\varphi}_l\left({\mathrm{SO}}_2\right)$,

where the *ϕ*_*i*_(PM_10_), *φ*_*l*_(SO_2_) and *η*_*k*_(NO_2_) are the basis functions and α_*i*_, *b*_*l*_ and *ω*_*k*_ are parameters. Next, the smooth of PM_10_, denoted by *f*(PM_10_), is converted into a smooth function of PM_10_ and SO_2_, denoted by *f*(PM_10_, SO_2_) (to make *f*(PM_10_) change smoothly with SO_2_), which can be achieved by allowing *α*_*i*_ to vary smoothly with SO_2_. Thus, we obtain

$$ {\alpha}_i\left({\mathrm{SO}}_2\right)=\sum \limits_{l=1}^L{b}_{il}{\varphi}_l\left({\mathrm{SO}}_2\right) $$

which results in

$$ f\left({\mathrm{PM}}_{10},{\mathrm{SO}}_2\right)={\sum}_{i=1}^I{\alpha}_i\left({\mathrm{SO}}_2\right){\phi}_i\left({\mathrm{PM}}_{10}\right)={\sum}_{i=1}^I{\sum}_{l=1}^L{b}_{il}\ {\varphi}_l\left({\mathrm{SO}}_2\right){\phi}_i\left({\mathrm{PM}}_{10}\right). $$

Similarly, we can now create a smooth function of PM_10_, SO_2_ and NO_2_ by allowing *f*(PM_10_, SO_2_) to vary smoothly with NO_2_. Therefore, with following the same reasoning as before, we obtain

$$ te\left({\mathrm{PM}}_{10},{\mathrm{NO}}_2,{\mathrm{SO}}_2\right)=f\left({\mathrm{PM}}_{10},{\mathrm{NO}}_2,{\mathrm{SO}}_2\right)=\sum \limits_{i=1}^I\sum \limits_{l=1}^L\sum \limits_{k=1}^K{v}_{ilk}{\eta}_k\left({\mathrm{NO}}_2\right){\varphi}_l\left({\mathrm{SO}}_2\right){\phi}_i\left({\mathrm{PM}}_{10}\right), $$

where *v*_*ilk*_ are the coefficients of basis functions of tensor product smooths.

According to the above description, it is clear that the complexity of the tensor product smooth function depends on the type of basis function and its dimensionality; therefore, it is essential to choose an appropriate basis function describing the relationship between an adverse outcome and air pollutants. In this study, we used the cubic spline basis function that is widely used to flexibly fit a potential nonlinear relationship.

To avoid the problem of collinearity and controlling the tradeoff between goodness of fit and model smoothness, we define the following penalized likelihood function to estimate parameters:

$$ l(v)+J\left(f\left({\mathrm{PM}}_{10},{\mathrm{NO}}_2,{\mathrm{SO}}_2\right)\right) $$

(and)

$$ J\left(f\left({\mathrm{PM}}_{10},{\mathrm{NO}}_2,{\mathrm{SO}}_2\right)\right)={\int}_{{\mathrm{PM}}_{10},{\mathrm{SO}}_2,{\mathrm{NO}}_2}\Big[{\lambda}_{{\mathrm{PM}}_{10}}{\left(\frac{\partial^2f}{\partial {\left({\mathrm{PM}}_{10}\right)}^2}\right)}^2+ $$

$$ \kern0.50em {\lambda}_{{\mathrm{NO}}_2}{\left(\frac{\partial^2f}{\partial {\left({\mathrm{NO}}_2\right)}^2}\right)}^2+{\lambda}_{{\mathrm{SO}}_2}{\left(\frac{\partial^2f}{\partial {\left({\mathrm{SO}}_2\right)}^2}\right)}^2\Big]d{\mathrm{PM}}_{10}d{\mathrm{NO}}_2d{\mathrm{SO}}_2, $$

where *l* (*v*) is the likelihood function, and *J*(*f*(PM_10_, NO_2_, SO_2_)) represents tensor product penalties. ${\lambda}_{{\mathrm{PM}}_{10}}$, ${\lambda}_{{\mathrm{NO}}_2}$ and ${\lambda}_{{\mathrm{SO}}_2}$ are smoothing parameters. Such parameters can be selected by Generalized Cross Validation (GCV). The estimates, denoted by ${\widehat{v}}_{ilk}$, are obtained by penalized iteratively re-weighted least squares which maximizes the penalized likelihood function. Furthermore, stratified analyses by educational attainment, sex and age were conducted respectively.

### Estimation of the combined effects

The combined effects of three air pollutants were measured by the combined rate ratio (RR). For the single-pollutant model and the three-pollutant non-interaction model, RR was calculated as the exponentiated sum of the products of each pollutant’s regression coefficient and the pollutant’s increment. For the three-pollutant interaction model, RR was calculated as the exponentiated sum of the following two parts: 1) the product of each pollutant’s regression coefficient and the pollutant’s increment and 2) the product of each interaction term coefficient and the difference value of the interaction term. The standard error of RR was estimated based on the variance-covariance matrix of pollutants’ coefficients. The detailed derivation process is described in the supplemental materials and uses the method proposed by Winquistet al.[13]. For the tensor production model, the RR is calculated as follows:

$$ {\boldsymbol{te}}^T\boldsymbol{\nu} =\boldsymbol{te}\left({{\mathrm{PM}}_{10}}^{{\mathrm{j}}^{\mathrm{th}}},{{\mathrm{NO}}_2}^{{\mathrm{j}}^{\mathrm{th}}},{{\mathrm{SO}}_2}^{{\mathrm{j}}^{\mathrm{th}}}\right)-\boldsymbol{te}\left({{\mathrm{PM}}_{10}}^{i^{\mathrm{th}}},{{\mathrm{NO}}_2}^{i^{\mathrm{th}}},{{\mathrm{SO}}_2}^{i^{\mathrm{th}}}\right), $$

where

$$ \boldsymbol{te}\left({{\mathrm{PM}}_{10}}^{{\mathrm{j}}^{\mathrm{th}}},{{\mathrm{NO}}_2}^{{\mathrm{j}}^{\mathrm{th}}},{{\mathrm{SO}}_2}^{{\mathrm{j}}^{\mathrm{th}}}\right)=\sum \limits_{i=1}^I\sum \limits_{l=1}^L\sum \limits_{k=1}^K{v}_{ilk}{\eta}_k\left({{\mathrm{NO}}_2}^{{\mathrm{j}}^{\mathrm{th}}}\right){\varphi}_l\left({{\mathrm{SO}}_2}^{{\mathrm{j}}^{\mathrm{th}}}\right){\phi}_i\left({{\mathrm{PM}}_{10}}^{{\mathrm{j}}^{\mathrm{th}}}\right); $$

$$ \boldsymbol{te}\left({{\mathrm{PM}}_{10}}^{i^{\mathrm{th}}},{{\mathrm{NO}}_2}^{i^{\mathrm{th}}},{{\mathrm{SO}}_2}^{i^{\mathrm{th}}}\right)=\sum \limits_{i=1}^I\sum \limits_{l=1}^L\sum \limits_{k=1}^K{v}_{ilk}{\eta}_k\left({{\mathrm{NO}}_2}^{i^{\mathrm{th}}}\right){\varphi}_l\left({{\mathrm{SO}}_2}^{i^{\mathrm{th}}}\right){\phi}_i\left({{\mathrm{PM}}_{10}}^{i^{\mathrm{th}}}\right). $$

Therefore, we can get *RR* =  *exp* (***te***^*T*^***ν***).

The values $\boldsymbol{te}\left({{\mathrm{PM}}_{10}}^{{\mathrm{j}}^{\mathrm{th}}},{{\mathrm{NO}}_2}^{{\mathrm{j}}^{\mathrm{th}}},{{\mathrm{SO}}_2}^{{\mathrm{j}}^{\mathrm{th}}}\right)$ and $\boldsymbol{te}\left({{\mathrm{PM}}_{10}}^{i^{\mathrm{th}}},{{\mathrm{NO}}_2}^{i^{\mathrm{th}}},{{\mathrm{SO}}_2}^{i^{\mathrm{th}}}\right)$ are the values of the tensor product smooths at the *j*^*th*^ and *i*^*th*^ percentiles of pollutants’ concentrations, respectively. The vector ***ν*** is the coefficient vector of the basis function of tensor product smooths. The vector ***te***^*T*^ is the transposed difference vector of basis functions of tensor product smooths at the *j*^*th*^ percentiles and the *i*^*th*^ percentiles of pollutants’ concentrations. The 95% confidence interval (CI) of RR was estimated by the following formula:

$$ 95\% CI=\left(\mathit{\exp}\left({\boldsymbol{te}}^T\boldsymbol{\nu} -{z}_{1-\alpha /2}\ {se}_{{\boldsymbol{te}}^T\boldsymbol{\nu}}\right),\mathit{\exp}\left({\boldsymbol{te}}^T\boldsymbol{\nu} -{z}_{1-\alpha /2}{se}_{{\boldsymbol{te}}^T\boldsymbol{\nu}}\right)\right), $$

$$ {se}_{{\boldsymbol{te}}^T\boldsymbol{\nu}}=\sqrt{{\boldsymbol{te}}^T\widehat{\varSigma}\boldsymbol{te}}, $$

where $\widehat{\Sigma}$ is the variance-covariance of the coefficients of the basis function and *α* =0.05.

To improve the readability of the results and better understand the excess burden due to air pollution, we calculated the excess risk (ER) of mortality by (RR-1)*100%, i.e., the percentage increase in mortality associated with air pollution.

To clarify the robustness of the lag selection for air pollutants, we conducted a sensitivity analysis to determine the effects of air pollution at lag 0–1 by using a 2-day moving average in models I-IV.

All analyses were performed using R software version 3.3.1, by using the “mgcv” used to fit the GAM.

## Results

**Fig. 1:**
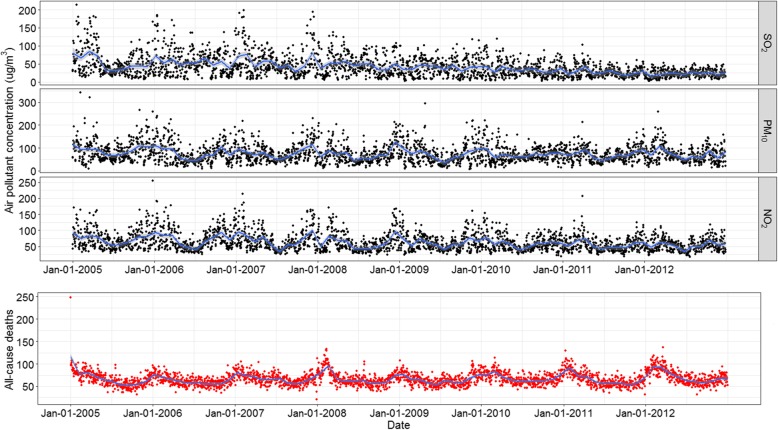
Time-series plots of air ambient pollutants and all-cause deaths during 2005–2012 in Guangzhou, China. PM_10_, particulate matter < 10 mm in aerodynamic diameter; SO_2_, sulfur dioxide; NO_2_, nitrogen dioxide

The total number of all-cause deaths was 193,715 during 2005–2012, with 66.34 cases per day. The daily mean temperature had an average of 22.5 ^°^C and ranged from 5.1 ^°^C to 34.2 ^°^C. The mean daily average concentrations of PM_10,_ SO_2_ and NO_2_ were 74.9 μg/m^3^, 41.0 μg/m^3^ and 62.4 μg/m^3^, respectively. The interquartile ranges (IQR) of three pollutants were 48.2 μg/m^3^, 33.7 μg/m^3^ and 33.3 μg/m^3^, respectively (Table 1). The time series plot of air ambient pollutants and all-cause deaths revealed a consistent seasonality; there were generally more deaths and higher concentrations of pollutants in the cool season (from May to October) than in the warm season (from November to April) (Fig. 1). The Spearman correlations of PM_10_ and NO_2_, PM_10_ and SO_2_, and NO_2_ and SO_2_ were 0.83, 0.60 and 0.65, respectively (*P* < 0.01).

**Table 1:** Summary statistics for daily number of deaths, daily air pollution concentrations and weather conditions in Guangzhou, China, 2005–2012

			Percentile			
Variables	Mean ± SD	Min	25th	50th	75th	Max
Daily number of deaths	66 ± 14	21	56	64	75	248
PM_10_ (μg/m^3^)	74.9 ± 39.6	7.6	46.4	66.9	94.6	342.3
SO_2_ (μg/m^3^)	41.0 ± 29.6	2.3	19.9	33.7	53.6	214.1
NO_2_ (μg/m^3^)	62.4 ± 27.8	16.7	42.1	55.6	75.4	254.7
Mean temperature (^°^C)	22.5 ± 6.3	5.1	18.0	24.2	27.7	34.2
Mean humidity (%)	72.9 ± 13.2	20.0	65.0	75.0	83.0	99.0
Mean pressure (hpa)	10,074 ± 70	9874	10,023	10,071	10,128	10,272

**Fig. 2:**
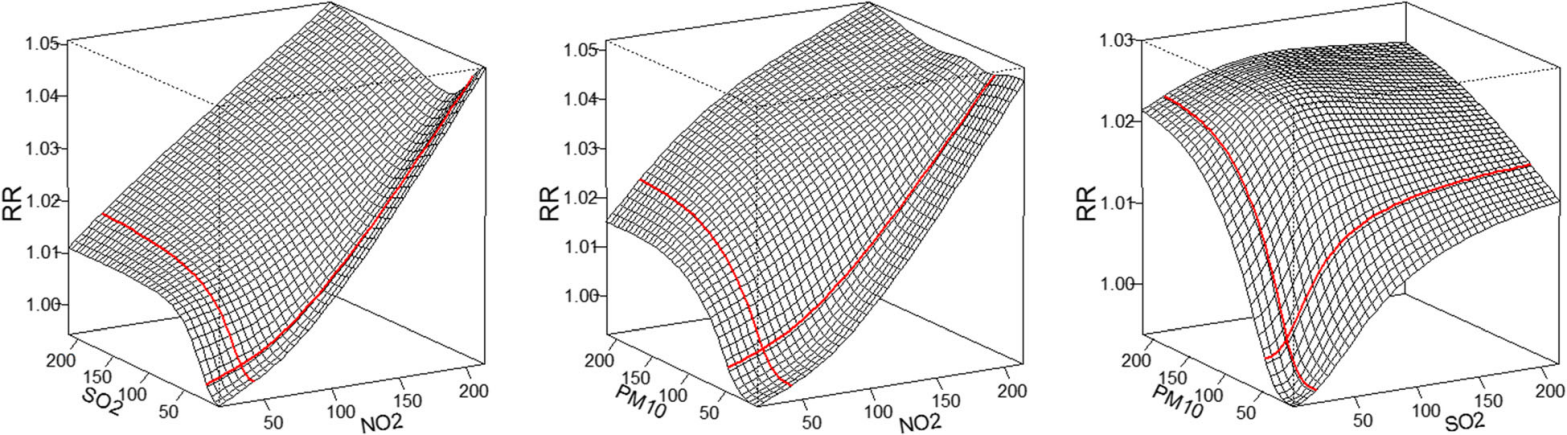
The combined effects of two pollutants on mortality, given the level of the remained pollutant fixed as the reference level. Red lines represent the exposure-response relationship between one pollutant and mortality when other two pollutants were fixed as the reference level (i.e. 25th percentile)

The tensor product model can produce a four-dimensional plot for the RR of mortality associated with the levels of three pollutants. To intuitively show the combined effects of air pollutants, the concentration of one of pollutants was fixed at the reference value (the 25th percentile was used in this study); then, a three-dimensional graph of RR of the other two pollutants can be drawn (Fig. 2). Generally, as the concentration of pollutants increased, a stronger combined effect of pollutants on mortality was observed, but the relationship was not completely linear or monotonic. The effect of one pollutant varied with the concentration of another pollutant. For example, the effect of PM_10_ increased significantly with the concentration of SO_2_ at low concentrations, but the curve of PM_10_ effect was relatively flat for high concentrations of SO_2_, which indicated that there was an interaction between pollutants.

**Fig. 3:**
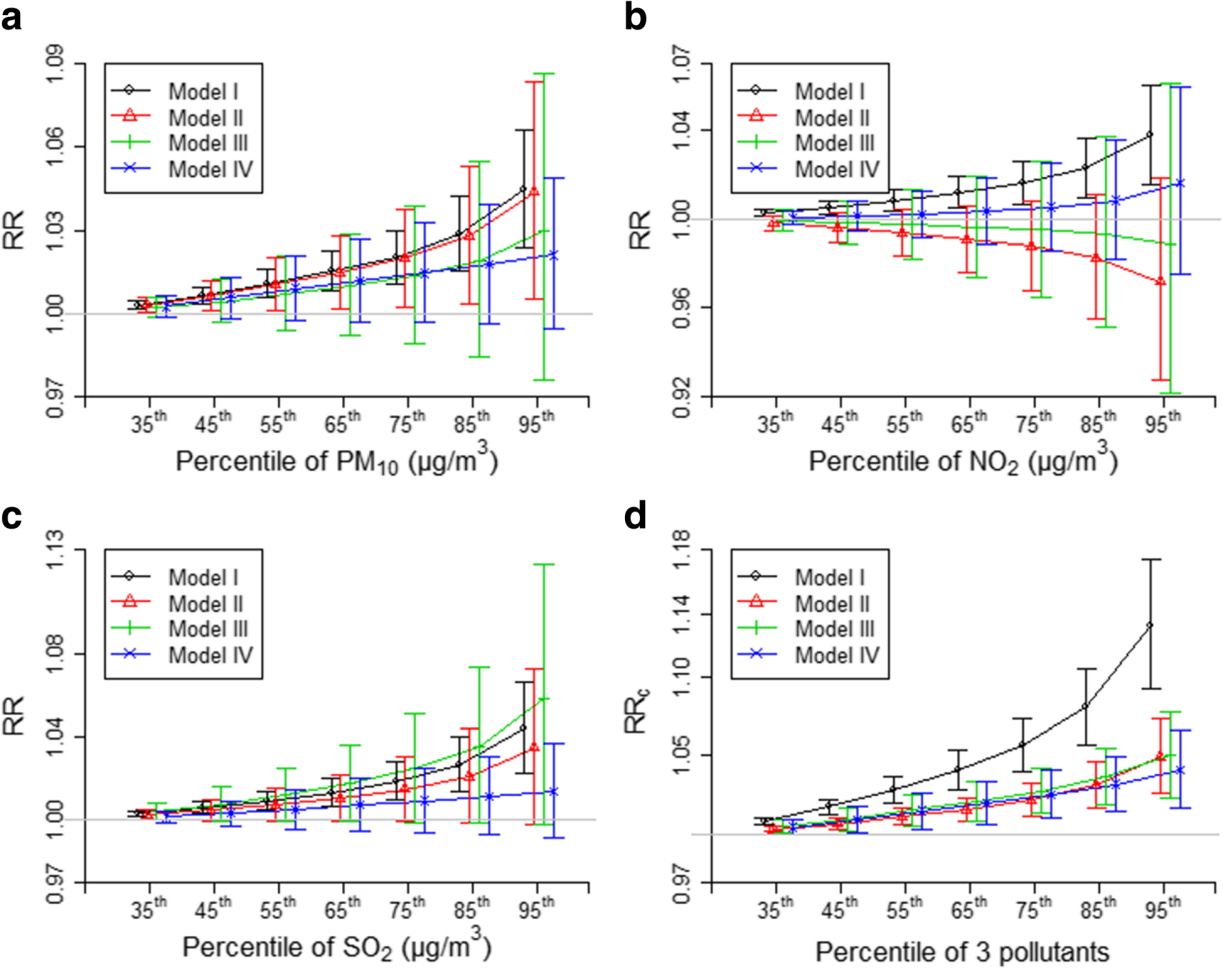
Mortality rate ratio (RR), estimated by models I-IV, for various levels of air pollution relative to the reference level. **a**-**c** show the exposure-response curves for PM_10_, NO_2_ and SO_2_, respectively, given the levels of other two pollutants fixed as their 25th percentiles. **d** shows the combined rate ratio (RR_c_) associated with the simultaneous increases in the levels of three pollutants. The horizontal lines in (**a**-**d**) indicate RR is equal to 1

Table 2 and Fig. 3 show the effects of air pollution estimated by four different models. The single-pollutant model (Model I) revealed that an IQR increase in the concentration of PM_10_, NO_2_ and SO_2_ was associated with increases in mortality of 2.03% (95% CI: 1.08–2.99%), 1.63% (95% CI: 0.69–2.59%) and 1.86% (95% CI: 0.94–2.78%), respectively. The level of NO_2_ was negatively associated with the risk of mortality if PM_10_ and SO_2_ were included simultaneously in the non-interaction model (models II-III). The combined effect of three pollutants estimated by the single-pollutant model (ER = 5.63, 95%CI: 3.96–7.34%) was much greater than those obtained from other three multi-pollutant models. The combined effect estimated by the three-pollutant non-interaction model (ER = 2.20, 95%CI: 1.18–3.23%) was slightly smaller than those from the interaction model (ER = 2.78, 95%CI: 1.35–4.23%) and the tensor product model (ER = 2.53, 95%CI: 1.03–4.01%). Fig. 3(a-c) shows that the tensor product model, compared to the three-pollutant non-interaction model and interaction model, produced more precise effect estimates (i.e., narrower 95%CI), especially for the main effects of single pollutants.

**Table 2:** Excess risk of mortality and 95% confidence intervals (%) associated with an IQR increment in air pollutant concentrations

Model^a^	PM_10_	NO_2_	SO_2_	Combined effects
I	2.03 (1.08–2.99)	1.63 (0.69–2.59)	1.86 (0.94–2.78)	5.63 (3.96–7.34)
II	1.98 (0.23–3.77)	− 1.24 (− 3.24–0.81)	1.47 (−0.10–3.07)	2.20 (1.18–3.23)
III	1.35 (− 1.11–3.88)	− 0.49 (− 3.51–2.61)	2.46 (− 0.11–5.10)	2.78 (1.35–4.23)
IV	1.45 (− 0.33–3.27)	0.53 (− 1.42–2.52)	0.92 (−0.65–2.52)	2.53 (1.03–4.01)

^a^Model I, II, III and IV denote the single-pollutant model, the three-pollutant non-interaction model, the three-pollutant interaction model and the tensor product model, respectively

Table 3 shows the results of stratified analyses by the tensor product model. There were a total number of 193,715 deaths during 2005–2012 in Guangzhou; 24.4, 56.4 and 92.6% of deaths were of those aged under 65, males and individuals with low educational attainment (illiterate or only primary education), respectively. Females, elderly people (65 years of age or above) and individuals with low educational attainment were more vulnerable to ambient air pollution exposure than were males, younger individuals and those with high educational attainment.

**Table 3:** The combined effects of three air pollutants on mortality by age, sex and educational attainment, estimated by the tensor product model

	Deaths (%)	ER^a^ (95%CI)
Age (years)		
< 65 years	47,303(24.4)	−1.09 (− 2.81–0.66)
≥ 65 years	146,412(75.4)	3.71 (1.96–5.50)
Gender		
Female	84,519(43.6)	3.58 (1.47–5.74)
Male	109,916(56.4)	1.50 (0.05–2.99)
Educational attainment		
Low	170,629(92.6)	2.82 (1.19–4.49)
High	13,734(7.4)	2.19 (−1.11–5.60)

^a^The excess risk (ER) of all-cause mortality associated with an IQR increment of concentrations of three pollutants

The sensitivity analysis for all models revealed that the effects at lag 0–1 were slightly larger than those at lag 0–2 (Additional file 1: Figure S1).

## Discussion

Most of previous studies of the health effects of air pollution are based on a single-pollutant model or include multiple pollutants in a model at the same time, thus ignoring the possibility of high correlation among pollutants that leads to the inflation of variance and the instability of model parameter estimation. The commonly used solution is stepwise regression and removing one of the highly correlated variables; however, this method may cause the loss of some important information. In this study, we proposed a quasi-Poisson regression with tensor product smooth to estimate the health effects of multiple pollutants. Overall, we observed that the combined effect of three pollutants (PM_10_, SO_2_ and NO_2_) gradually increased along with concentration of pollutants. Moreover, there was a complex interaction among the pollutants. The results of the stratified analysis showed that elderly individuals, females and people with low educational attainment were more vulnerable to air pollution.

The single-pollutant model, a common method of assessing health effects of air pollution, revealed that the levels of each single pollutant (PM_10_, NO_2_ and SO_2_) had a significantly positive relationship with the risk of all-cause deaths, with ER values associated with IQR increase in the concentration of lag 0–2 of 2.03, 1.63 and 1.86%, respectively. We observed that the effect estimates of PM_10_ and NO_2_were greater at lag 0–1, with ER values per IQR increase of 2.13, 1.73 and 1.79% (i.e., 0.44, 0.52 and 0.53% per 10ug/m^3^ increase). Consistently, Wu et al. reported that ER of mortality associated with IQR increase in PM_10_ in Guangzhou was 1.77% (95%CI: 0.37–3.18%) in 2006–2009 and 2.03% (95%CI: 0.79–3.30%) in 2010–2013, and the effect estimates were very similar after adjusting for NO_2_ or SO_2_ [35]. The estimate of the PM_10_ effect was also very similar to that reported in 90 American cities and in 38 Chinese cities (i.e., 0.5 and 0.44% per 10ug/m^3^ increase), while the effects of SO_2_ and NO_2_ we observed were smaller than the estimates in four Asian cities (i.e., 1.00 and 1.23% per 10μg/m^3^ increase) [34, 36].

The three-pollutant models produced abnormal health impact estimates of single pollutants (e.g., a negative effect of NO_2_) and w19pt?>The above phenomenon was due to strong collinearity among pollutants with Spearman correlation coefficients of 0.60–0.83, which caused variance inflation of the regression parameters and bias in the statistical inference [11]. As to the combined effects of three pollutants, it was clear that the additive effect of a single pollutant based on the single-pollutant model was much higher than the combined effect estimated by other three multi-pollutant models. The reasons are that the single pollutant model does not take into account the high correlation between pollutants, and one air pollutant may be a substitute for another air pollutant or the mixture of air pollutants [37].

The most innovative point of this study is that the tensor product quasi-Poisson regression model was, for the first time, applied to assess the combined effect of several pollutants on adverse health outcome in the presence of multi-collinearity and to explore the complex interaction of several pollutants. Several methods that were proposed in the past made additional assumptions of the existence of a linear or specific functional relationship between atmospheric pollutants and the response variable or only explored the first order interaction; in contrast, the GAM with tensor product smooth not only relaxes those assumptions but also flexibly explores the nonlinear effect and the complex interaction of multiple pollutants. Specially, this method gives a highly visual depiction of the relationship between all-cause deaths and air pollutants, which may allow analysts to discover more undetectable characteristics of data than would be possible with the use of a simple model, and provide a new approach to comprehensive evaluation of health effects of multiple pollutants.

In this study, the tensor product model was also used to identify subpopulations that were more susceptible to exposure to a mixture of multiple pollutants. Some studies that assessed the health effect of single pollutants revealed that females were more susceptible than males to adverse effects of ambient air pollution [38]. Kan et al. observed that the effects of PM_10_ on mortality were almost twice as strong in females [39]. The reasons for sexual differences were unclear and deserved to be further investigated. We observed greater combined effects of multiple pollutants in females than in males. Residents with low educational attainment were more sensitive to exposure to ambient air pollution than those with high educational attainment, which was consistent with previous studies [39, 40]. Most individuals with low educational attainment experience higher financial risk and, therefore, potentially also experience inferior living conditions and inadequate healthcare. Additionally, individuals with low educational attainment were more likely to be occupationally exposed to air pollution [41].

Our study has some limitations. Although the GAM with tensor product smooth is flexible enough to explore the relationship between adverse health outcome and air pollutants, the “curse of dimensionality” occurs when the number of variables in the tensor product smooth increases, which is inherent in many flexible models [23]. The total dimensionality is the product of dimensionalities of each pollutant; thus, a large sample size is needed to accurately estimate parameters, and the computation is time-consuming. Moreover, it is difficult to interpret results of the model in high-dimensional spaces. We assessed health effects of three main pollutants to demonstrate the methods; however, other pollutants, such as ozone and PM_2.5_ that might be related to mortality were not considered in this study because of data unavailability during the study period.

## Conclusions

Ambient air pollution has a significant impact on mortality. Our findings indicate that there exists a high correlation and a complex interaction among air pollutants. The single-pollutant model is unable to accurately estimate the combined effects of multiple pollutants. The simple multi-pollutant model with or without the first-order interaction term may yield misleading results when attributing health effects to individual pollutants. This study provides a novel method, called a tensor product quasi-Poisson model, for assessing the health effects of multiple pollutants, which can be applied in various epidemiologic studies to determine the combined effects of environmental, social and individual risk factors, especially if such factors are highly correlated and have nonlinear health effects.

## Additional file

Additional file 1: **FigureS1.** Excess risk of mortality and 95% confidence intervals (%) associated with an IQR increment in moving average lag of 0–1 and 0–2 of air pollutants. Lag0-1and lag0–2 refer to moving average of the current day and preceding 1 day and 2 days. I-IV denote the models I-IV. (TIFF 551 kb)

## Abbreviations

BKMR: Bayesian kernel machine regression
BMA: Bayesian model averaging
CI: Confidence interval
df: Degree of freedom
ER: Excess risk
GAM: Generalized additive model
HOD: Holiday
IQR: Interquartile-range
NO_2_: Nitrogen dioxide
ns: Nature cubic spline
PACF: Partial autocorrelation function
PCA: Principal component analysis
PLSR: Partial least-square regression
PM_10_: Particulate matter less than 10 μm in aerodynamic diameter
RR: Rate ratio
SO_2_: Sulfur dioxide
te: Tensor product smooths
