# Supplementary figures and images for: A tensor product quasi-Poisson model for estimating health effects of multiple ambient pollutants on mortality
Source: Environ Health. 2019 Apr 24;18:38. doi: 10.1186/s12940-019-0473-7 (PMC6480885; doi:10.1186/s12940-019-0473-7)

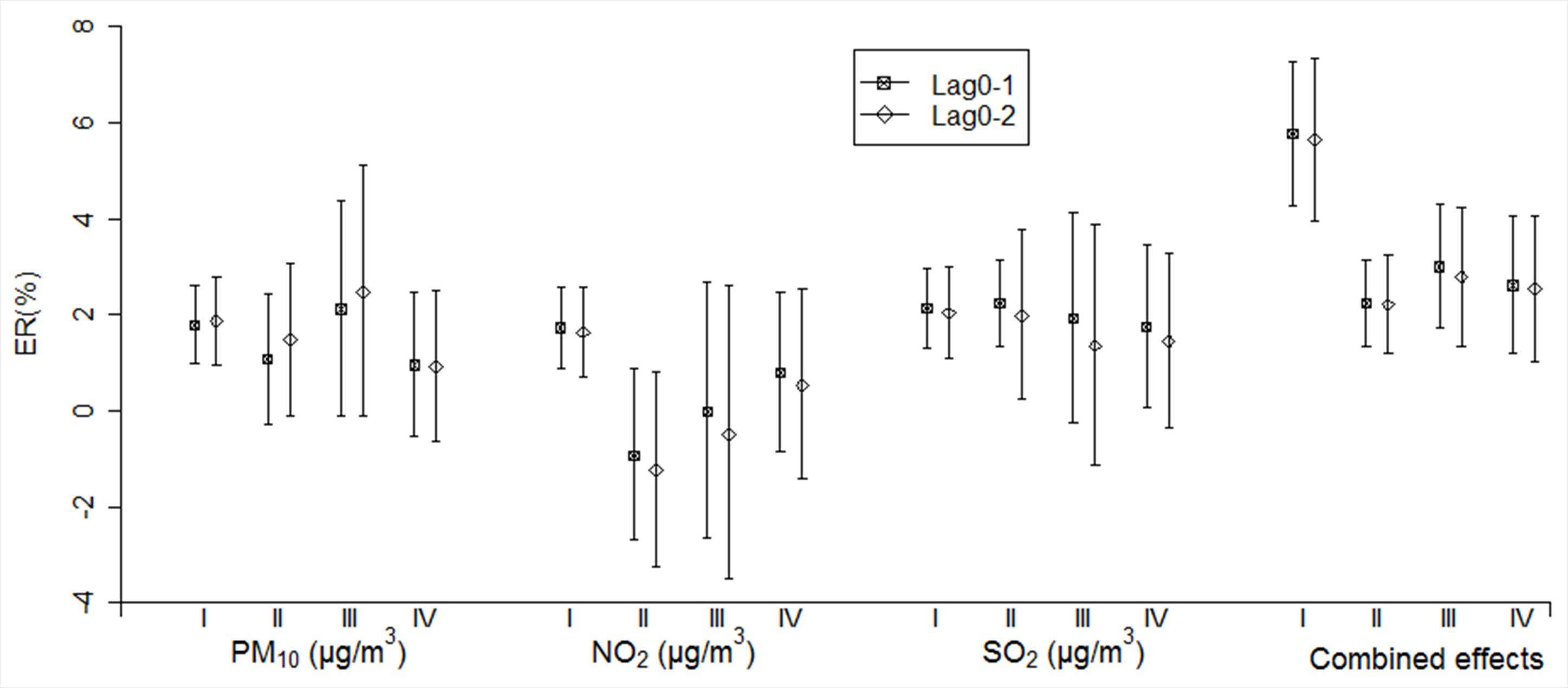

Supplement: Supplementary file 1 — FigureS1. Excess risk of mortality and 95% confidence intervals (%) associated with an IQR increment in moving average lag of 0–1 and 0–2 of air pollutants. Lag0-1and lag0–2 refer to moving average of the current day and preceding 1 day and 2 days. I-IV denote the models I-IV. (TIFF 551 kb) [file 12940_2019_473_MOESM1_ESM.tiff]
